# Changes in serum metal concentrations following high-intensity interval training: A 16-week pilot study

**DOI:** 10.1016/j.clinsp.2025.100802

**Published:** 2025-10-14

**Authors:** Guilherme da Silva Rodrigues, Natalia Yumi Noronha, Jhennyfer Aline Lima Rodrigues, Andressa Crystine da Silva Sobrinho, Jonas Benjamim, Luísa Maria Diani, Isabela Harumi Yonehara Noma, Fernando Barbosa Júnior, Lígia Moriguchi Watanabe, Carla Barbosa Nonino, Carlos Roberto Bueno Júnior

**Affiliations:** a Department of Internal Medicine, Faculdade de Medicina de Ribeirão Preto, Universidade de São Paulo, Ribeirão Preto, SP, Brazil; b Institute for Physical Activity and Nutrition, School of Exercise and Nutrition Sciences, Deakin University, Geelong, Australia; c Department of Clinical and Toxicological Analyses, Faculdade de Ciências Farmacêuticas da Universidade de São Paulo, São Paulo, SP, Brazil; d Department of Clinical Analyses, Toxicology and Food Science, Faculdade de Ciências Farmacêuticas de Ribeirão Preto, Universidade de São Paulo, Ribeirão Preto, SP, Brazil; e Department of Health Sciences, Faculdade de Medicina de Ribeirão Preto, Ribeirão Preto, SP, Brazil; f Escola de Educação Física de Ribeirão Preto da Universidade de São Paulo, Ribeirão Preto, SP, Brazil

**Keywords:** Toxic metals, Excretion of metals, Exercise intervention, Aerobic exercise, Metabolic health

## Abstract

**Introduction:**

High-Intensity Interval Training (HIIT), involving brief bouts of intense exercise with rest or low-intensity intervals, benefits cardiovascular, metabolic health, and body composition. However, its impact on serum metal concentrations in adults remains unclear. This study investigated the effects of a 16-week HIIT protocol on serum metal levels.

**Methods:**

Adults over 18-years-old with a Body Mass Index (BMI) above 25 kg/m^2^. Participants trained for 40-minutes three times per week, with two supervised and one unsupervised session. Each session included a 10-minute warm-up at 70 % of maximum Heart Rate (HRmax), followed by four 4-minute intervals at 90 % HRmax, interspersed with three 3-minute active pauses at 70 % HRmax. Before and after the 16-week intervention, anthropometric and biochemical parameters were assessed.

**Results:**

Fifteen participants [mean (SD) age: 42 (6); BMI: 30.6 (2.7)] completed the study. Significant reductions were observed in both toxic (e.g., mercury, lithium, lead, nickel, aluminum) and essential metals (e.g., manganese): mercury (*p* = 0.025, Cohen’s *d* = 0.58), lithium (*p* = 0.009, *d* = 1.01), aluminum (*p* = 0.025, *d* = 0.49), manganese (*p* = 0.009, *d* = 0.96), nickel (*p* = 0.025, *d* = 0.58), and lead (*p* = 0.025, *d* = 0.40). A negative correlation was found between the change in hip circumference and manganese (*r* = -0.538; *p* = 0.047), and a positive correlation with nickel (*r* = 0.600; *p* = 0.023).

**Conclusion:**

These findings suggest potential associations between changes in metal concentrations and anthropometric outcomes following HIIT.

## Highlights

• HIIT for 16-weeks reduced both essential and toxic serum metal levels.
• High-intensity training altered the body’s metal accumulation profile.
• Manganese reduction was linked to lower hip circumference post-HIIT.
• Exercise-induced metal shifts may relate to metabolic health outcomes.

## Introduction

High-Intensity Interval Training (HIIT) has been extensively studied over recent decades due to its significant benefits for cardiovascular, metabolic health, and body composition.^1^ This type of training is characterized by short periods of intense exercise alternated with periods of rest or low-intensity exercise, making it an efficient and less time-consuming alternative compared to traditional continuous aerobic exercise methods.^2^ Although HIIT can improve the body's antioxidant capacity,^3^, ^4^, ^5^ there is no direct evidence that this type of training helps eliminate heavy metals from the body. The ability of physical exercise in general, and HIIT in particular, to influence the mobilization and excretion of these heavy metals is an emerging area of research.

Heavy metals such as lead, mercury, cadmium, and arsenic can accumulate in the human body fat. They are introduced into the environment primarily through industrial, agricultural, and other anthropogenic processes. Chronic exposure to these metals is associated with neurological dysfunctions, cardiovascular diseases, kidney and liver damage, and cancer.^6^ Poisoning occurs mainly through inhalation, ingestion, or dermal contact, with symptoms varying according to the type of metal and level of exposure. For example, mercury can cause tremors, vision problems, behavioral changes, and permanent brain damage, while lead is related to cognitive problems in children, anemia, and hypertension.^7^

There is evidence that physical exercise can help reduce the toxic metals burden in the body.^8^ A recent literature review showed that cognitive and motor exercises can improve brain function and attenuate neurodegeneration in patients with heavy metal-induced neurotoxicity.^9^ Research has shown that the body has the ability to excrete nickel, lead, copper, and arsenic through sweat, and eliminating heavy metals from the body through dynamic exercise may be more effective than eliminating them through static exposure to a hot environment.^10^ Kuan et al.^10^ demonstrated that regular exercise can increase the excretion of these metals through sweat and urine, improving kidney function and metabolic efficiency. However, the specific effectiveness of HIIT in the excretion of heavy metals is still underexplored, highlighting the need for more research in this area.

This study investigates the impact of 16-weeks of HIIT on toxic metals concentration in adults. The existing literature provides a solid foundation for this investigation, highlighting the benefits of HIIT in various populations. Studies have shown that HIIT can significantly improve physical and mental health in adults with cardiovascular diseases, reducing waist circumference and improving lipid and glycemic.^11^^,^^12^ Furthermore, a systematic meta-analysis highlighted that HIIT can reduce body fat indicators in overweight and obese adults, suggesting that HIIT is an efficient component for weight management programs.^13^

In addition to improvements in body composition, HIIT has also demonstrated efficacy in reducing cardiometabolic risk factors. A 16-week intervention study found significant improvements in cardiorespiratory fitness and blood pressure among individuals with metabolic syndrome.^14^ These findings are corroborated by another study that reported reductions in waist circumference and improvements in lipid profiles after 2-weeks of HIIT in female university students.^15^ Recent studies indicate that physical exercise may help reduce the concentration of these metals in the body, possibly due to improved kidney function and overall metabolism.^10^^,^^16^ However, research in this area is still limited, and more studies are needed to elucidate the mechanisms involved.

Therefore, this study aims to investigate the effects of a 16-week HIIT protocol on serum metal concentration in adults, addressing significant gaps in the current literature. While many studies have explored the benefits of HIIT on metabolic health and body composition, few have examined its impact on reducing heavy metals. The hypothesis is that HIIT may contribute to reductions in toxic metal levels in the body, which could be relevant for improving metabolic health and mitigating risks associated with prolonged exposure. This study is expected to generate preliminary insights that may inform future interventions aimed at managing toxic metal accumulation.

## Material and methods

This pilot study followed a 16-week longitudinal observational design (pre-post intervention without a control group), in line with STROBE guidelines. This study was conducted in accordance with the recommendations of the Declaration of Helsinki and Resolution n° 466, dated December 12, 2012, of the National Health Council of Brazil. The study was approved by the Research Ethics Committee of the School of Physical Education and Sport at the University of São Paulo, with registration number CAAE 37,573,114.6.0000.5659. All participants signed the informed consent form to participate in the research.

The authors included adults (> 18-years-old) with a BMI above 25 kg/m^2^. The potential participants should not have limitations for physical exercise due to the need to complete the 16-week training. Participants were not included if they presented a previous acute myocardial infarction, uncontrolled hypertension (systolic > 160 mmHg or diastolic blood pressure > 105 mmHg), planned surgeries during the study period, and alcohol or drug abuse. Men and women with risk factors for metabolic syndrome, according to the criteria of the International Diabetes Federation, were eligible for the study.^17^ Study participants were physically inactive at baseline, as assessed by the International Physical Activity Questionnaire (IPAQ).^18^ The authors adopted physically inactive participants so that all participants would have a similar fitness level prior to the intervention.

### Sample size

To reinforce the statistical reliability of the findings, a power analysis was conducted using G*Power software. A post hoc calculation based on the significant effect observed for manganese (Cohen’s *d* = 0.96) demonstrated that the present study reached a power of 93.2 % with 15 participants, suggesting that the study was adequately powered to detect large effects.

In parallel, an a priori simulation estimated that 34 participants would be required to detect a moderate effect (*d* = 0.5) with 80 % power and α = 0.05 in a similar paired-sample design. This supports the interpretation of robust findings where significant effects were observed and highlights the importance of increasing sample size in future confirmatory studies.

### Training protocol

The physical training was conducted on TRG motorized treadmills (Progress T6, Brazil). Training sessions were held at the same time of day based on the participants’ availability. The first training session was used to determine the treadmill speed and the need for incline, using the percentage of maximum heart rate (HRmax) and the rate of perceived exertion (Borg scale 6‒20) as references.^17^ Since the HIIT protocol was standardized, with intensities defined in relation to HRmax, the participant needed to reach the target Heart Rate (HR) of the training previously assessed. With some participants throughout the 16-weeks of intervention, the authors adopted the incline in the first weeks of intervention, while others needed a longer time, but it is worth mentioning that all 15 study participants reached the target intensity (HR or RPE).

The HIIT protocol adopted in the study was the 4 × 4 protocol. Participants trained for 40 minutes, three times a week, with two supervised sessions and one unsupervised session, which could be performed anywhere appropriate to maintain the training intensities.^17^^,^^19^ The session was divided as follows: participants performed a 10 minute warm-up at 70 % HRmax. After warming up, participants completed four 4-minute intervals at 90 % HRmax, following an interspersion with three active pauses of 3 minutes each at 70 % HRmax. As a cool-down and recovery, the final five minutes were used.^17^^,^^19^

### Assessments

At the baseline, the participants were assessed with measurements of weight (kg), height (cm), waist and hip circumferences (cm). Later, the authors calculated the BMI (kg/m^2^). After 16-week of HIIT intervention protocol, the same variables were assessed by the same researcher.^17^ Blood samples were collected through venipuncture on the left arm after a 12-hour fasting period. The authors followed the same timing protocol as the anthropometric assessments, with blood drawings occurring between 7:30 and 9:00 AM. In this study, the authors measured blood glucose (mmoL/L), triglycerides (mmoL/L), total cholesterol (mmoL/L), Low-Density Lipoprotein (LDL-c) (mmoL/L), and High-Density Lipoprotein (HDL-c) (mmoL/L) levels.

Blood samples for metal analysis were collected under the same standardized conditions as described for biochemical assessments: after a 12-hour fasting period and between 7:30 and 9:00 AM. Both baseline and post-intervention samples were collected on scheduled assessment days, with participants at rest and not having performed physical exercise on the same day prior to sampling. Therefore, the influence of exercise-induced hemoconcentration was minimized, and no additional correction for plasma volume shifts was necessary.

The analyses were performed by a qualified professional using the BT 3000 plus autoanalyzer from Wiener Lab.^17^ Total serum concentrations of metals (²⁰²Hg, ²⁷Al, ⁷⁵As, ¹¹¹Cd, ²⁰⁸Pb, ⁶⁰Ni, and ⁷Li) were determined using Inductively Coupled Plasma Mass Spectrometry (ICP-MS), equipped with a dynamic reaction cell. Samples were diluted at a 1:20 ratio with a solution containing 0.005 % v/v Triton® X-100 and 0.5 % v/v distilled HNO₃. Calibration standards ranged from 0.5 to 50 μg/L, and rhodium (10 mg/L) was used as an internal standard. To ensure analytical quality, certified reference materials (QMEQAS07B06 and QMEQAS07B03, L′Institut National de Santé Publique du Quebec) were analyzed before and after every 10 samples.

Recovery rates for all elements ranged from 90 % to 99 %, with intra-assay precision below 8 %, and method detection limits of 0.5 μg/L, in agreement with established validation parameters previously reported by the studied group.^20^ These metrics confirm the reliability and reproducibility of the ICP-MS methodology used in this study.

### Statistical analysis

The authors performed visual inspection of outliers using box plots created on Microsoft Excel®, and then the Shapiro-Wilk statistical test was used to test data normality distribution. Statistical analyses were performed on SPSS v.22 software using an intention-to-treat approach. Pre vs. post-intervention data were analyzed using the student’s *t*-test or the non-parametric Mann-Whitney *U* to perform comparisons between the pre- and post-16-week intervention moments. It is worth noting that the authors adopted significance levels of *p* < 0.05 for the analyses used in the study. Variable values are presented with mean and Standard Deviation (SD). Paired *t*-tests were used to compare metal concentrations pre- and post-intervention. To control multiple comparisons, p-values were adjusted using both the Bonferroni and the Benjamini-Hochberg False Discovery Rate (FDR) methods (RStudio version 4.4.0). Given the exploratory nature of the analysis and the risk of increased type II error with conservative approaches, FDR-adjusted p-values were adopted as the primary criterion for statistical significance (*p* < 0.05). Effect sizes were calculated using Cohen’s *d* for paired samples, with values of 0.2, 0.5, and 0.8 interpreted as small, moderate, and large effects, respectively (RStudio version 4.4.0).

To assess the relationships between pre- and post-exercise changes, the authors conducted a Pearson correlation analysis (RStudio version 4.4.0). Changes were quantified as deltas, calculated by the formula (post-pre) for each variable. The authors focused on variables that exhibited significant differences between post- and pre-exercise measurements. These variables included clinical variables such as hip circumference, as well as serum concentrations of aluminum, mercury, lithium, manganese, nickel, and lead. Deltas were used to assess correlations between changes in clinical variables and changes in serum metal concentrations after a High-Intensity Interval Training (HIIT) intervention. It is worth noting that the authors adopted significance levels of *p* < 0.05 for the analyses used in the study.

## Results

Fifteen participants [mean (SD) age: 42y (6); height (m): 1.6 (0.1)] completed the study protocol. When comparing pre- and post-intervention results after the 16-week HIIT 4 × 4 training, a statistically significant difference was observed in the hip circumference variable (*p* = 0.001), with a reduction in measurements after the training (Table 1).

**Table 1:** Anthropometric and blood pressure variables pre- and post- 16-weeks of HIIT training (*n* = 15).

Variables	Pre	Post	p-value	Cohen's *d*
Weight (kg)	77.1 (8.1)	75.7 (7.8)	0.934	
BMI (kg/m^2^)	30.8 (2.7)	30.2 (2.5)	0.327	
Waist circumference (cm)	90.9 (5.4)	90.1 (5.7)	0.859	
Hip circumference (cm)	111.4 (6.3)	109.4 (6.1)	0.001^a^	1.04
Systolic blood pressure (mmHg)	118.3 (9.2)	110.6 (11.7)	0.189	
Diastolic blood pressure (mmHg)	72.2 (4.6)	72.4 (4.1)	0.877	

BMI, Body Mass Index; cm, centimeters; mmHG, millimeters of mercury; kg, kilogram; kg/m^2^, weight (kg) by height squared (meters).

a Student’s *t*-test or Mann-Whitney non-parametric approach as appropriate (*p* < 0.05). Effect sizes for pre- and post-intervention comparisons were calculated using Cohen’s *d* for paired samples. According to conventional thresholds, *d* = 0.2 indicates a small effect, *d* = 0.5 a moderate effect, and *d* = 0.8 or above a large effect.

The training protocol used in this study did not show significant differences in biochemical variables (Table 2).

**Table 2:** Biochemical variables pre- and post-16-weeks of HIIT training (*n* = 15).

Variables	Pre	Post	p-value
Glycemia (mmoL/L)	4.9 (0.5)	5.0 (0.6)	0.866
Triglycerides (mmoL/L)	1.1 (0.4)	1.0 (0.4)	0.153
Total Cholesterol (mmoL/L)	4.8 (0.5)	5.1 (0.6)	0.184
HDL-c (mmoL/L)	1.2 (0.2)	1.3 (0.2)	0.878
LDL-c (mmoL/L)	3.1 (0.6)	3.4 (0.5)	0.769

HDL-c, High Density Lipoprotein Cholesterol; LDL-c, Low Density Lipoprotein Cholesterol; mmoL/L, millimoles per litre.

* Student’s *t*-test or Mann-Whitney non-parametric approach as (*p* < 0.05).

**Fig. 1:**
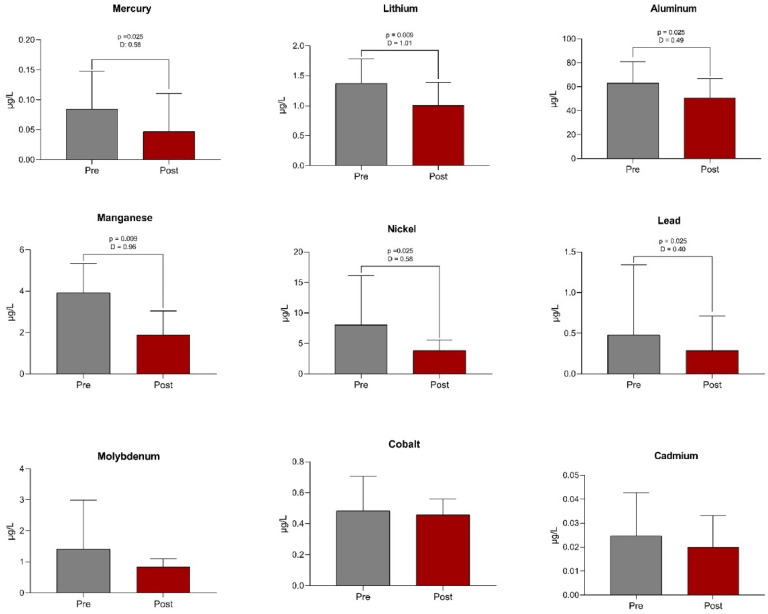
Pre- and post- 16-weeks HIIT comparisons of both toxic and essential metals measured in blood (*n* = 15). μg/L, microgram per liter; D, Cohen's *d*; p, p-value. Comparisons were performed using Student’s *t*-test or the non-parametric Mann-Whitney test, as appropriate. p-values were adjusted for multiple comparisons using the Benjamini-Hochberg False Discovery Rate (FDR) method. Effect sizes for pre- and post-intervention comparisons were calculated using Cohen’s *d* for paired samples. According to conventional thresholds, *d* = 0.2 indicates a small effect, *d* = 0.5 a moderate effect, and *d* = 0.8 or above a large effect. Significance was set at FDR-adjusted *p* < 0.05.

The present data showed significant reductions in the blood concentration of both toxic and essential metals in the study population. After 16-weeks of training, the present data revealed reductions in the concentrations of mercury (*p* = 0.022), lithium (*p* = 0.002), aluminum (*p* = 0.018), manganese (*p* = 0.003), nickel (*p* = 0.014), and lead (*p* = 0.025) (Fig. 1). Although Bonferroni-adjusted p-values exceeded the conventional significance threshold (*p* > 0.05), all six metals remained statistically significant after FDR correction, supporting the robustness of the findings.

**Fig. 2:**
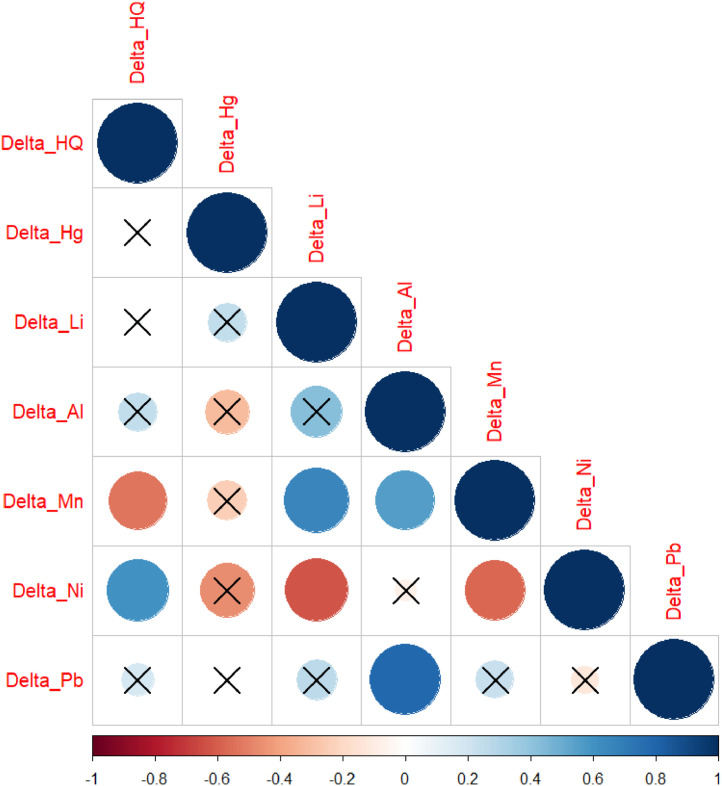
Pearson’s correlation with Delta values of variables found to be significant in the study (*n* = 15). Delta_HQ, Variation of hip circumference; Delta_Hg, Variation of mercury level; Delta_Li, Variation of lithium levels; Delta_Al, Variation of aluminum levels; Delta_Mn, Variation of manganese levels; Delta_Ni, Variation of nickel levels; Delta_Pb, Variation of lead levels. Pearson’s correlation significant if *p* < 0.05.

Pearson’s correlation analysis revealed significant correlations between the delta values of certain variables. Notably, the authors observed a negative correlation between the delta of Manganese and the delta of Hip Circumference (*r* = −0.538, *p* = 0.047), indicating that as the Manganese levels decreased, the Hip Circumference also tended to increase (Fig. 2).

Additionally, a positive correlation was found between the delta of Nickel and the delta of Hip Circumference (*r* = 0.600, *p* = 0.023), suggesting that decreases in Nickel levels were associated with decreases in Hip Circumference (Fig. 2). These results highlight potential relationships between changes in specific serum metal concentrations and anthropometric measurements following HIIT exercise.

## Discussion

This study aimed to investigate the effects of 16-weeks of High-Intensity Interval Training (HIIT) on serum metal concentrations in overweight adults. The results demonstrated a significant reduction in hip circumference, as well as in the concentrations of six metals: mercury, lithium, aluminum, manganese, nickel, and lead. Additionally, significant correlations were observed between changes in hip circumference and serum levels of manganese (negative correlation) and nickel (positive correlation), suggesting that the training protocol may influence not only anthropometric parameters but also the systemic metal profile.

HIIT is repeated bouts of short to long duration exercise completed at an intensity greater than the anaerobic threshold, interspersed with recovery periods. This method is the rapid metabolic adaptations promoted and a shorter time allocated to the training.^21^^,^^22^ After 16-weeks of HIIT training, these results showed a decrease in hip circumference and reductions in both toxic and essential metals concentrations.

This result is expected, according to other studies that showed differences in weight, waist circumference, hip circumference, and other biochemical parameters after 8-weeks of exercise.^23^ Also, they found decreases in body mass, BMI, waist and hip circumference after training.^24^ The present results show a reduction in both toxic and essential metals, such as mercury, lithium, aluminum, manganese, and lead.

Mercury and lead are toxic metals that are mostly involved in causing health effects. Lead and mercury are deleterious to human health if accumulated in the body. On the other hand, manganese is required by the human body; however, it may be toxic in higher concentrations. Pb is able to inhibit calcium actions and the ability of Pb to react with proteins. Pb can be incorporated into minerals instead of calcium, and interfere with normal actions.^25^

Lithium also has a harmful effect ‒ one review showed an association of lithium with increased risk of several diseases and outcomes.^26^ Manganese is an essential trace metal important for normal cell function and metabolism. However, it can be toxic in high concentrations, such as excessive dietary exposure to Mn, which can lead to accumulation in the brain, and consequently neurotoxicity.^27^ In this study, the authors observed that as manganese levels decreased, hip circumference tended to increase, highlighting the importance of maintaining adequate levels of this essential metal during HIIT. While reduced Mn levels may reflect decreased adiposity, clinicians should monitor essential metal status during HIIT interventions to prevent deficiency.

The authors found that HIIT is able to decrease toxic metals, avoiding several conditions due to an excess of these metals. The present study also demonstrated the correlations between Mg and Hip circumference, as shown by another study that blood manganese levels were directly associated with waist circumference.^28^ The present results presented a relation between nickel and hip circumference, which was also found in another study, where they detected the relationship between nickel exposure and waist in the general population of the United States.^29^

Several studies have investigated the correlation between toxic metals and obesity. These studies have observed that individuals with obesity have increased exposure to lead and mercury, particularly in subcutaneous fat deposits. Researchers have suggested that Hg exposure is associated with abdominal and subcutaneous fat accumulation, while Pb exposure is linked to differential fat accumulation. Moreover, there is a biologically plausible explanation for the involvement of toxic metals in the development of obesity.^30^

Another study has proposed that cumulative exposure to metal mixtures may contribute to both obesity and its related chronic conditions. This research revealed an association between cumulative exposure to mixtures of 18 toxic metals and obesity and its comorbidities among US adults.^31^ Furthermore, a positive association has been established between arsenic, cadmium, iron, lead, mercury, and fatty liver disease.^32^

Although these findings indicate a reduction in circulating metal concentrations after HIIT, it is important to acknowledge that other studies have suggested that intense physical exercise can transiently increase oxidative stress and potentially mobilize metals from tissue stores into circulation.^33^^,^^34^ For instance, elevated levels of reactive oxygen species during and after exercise may increase lipid peroxidation and alter metal-binding proteins,^35^^,^^36^ thereby releasing metals such as cadmium or mercury into the bloodstream. These findings highlight the dual nature of exercise: while beneficial for metabolic health, it can also promote temporary shifts in metal homeostasis, especially under high-intensity protocols.^37^^,^^38^ Therefore, future studies should include serial post-exercise measurements and assessments of metal excretion (e.g., in urine or sweat) to clarify whether the observed reductions reflect true clearance or redistribution dynamics.

This study has several limitations that should be considered when interpreting the findings. First, the absence of a control group limits the ability to draw definitive causal inferences regarding the effects of HIIT on metal concentrations. Additionally, although participants were instructed to follow the unsupervised session with the same intensity as the supervised ones, the authors cannot ensure full adherence to the protocol during these sessions. Moreover, the small sample size reduces statistical power and may limit the generalizability of the findings to broader populations.

Despite these limitations, this study offers important preliminary insights into the potential of HIIT to influence toxic metal levels. The protocol was well tolerated, and the use of a validated, high-intensity training model supports the reproducibility of the approach. Future studies should incorporate a randomized controlled design, larger and more diverse samples, and mechanistic assessments such as metal excretion in sweat or urine to further elucidate the physiological pathways underlying the observed effects. Until then, these findings should be interpreted as hypothesis-generating.

## Conclusion

The present findings suggest a reduction in the concentrations of both toxic and essential metals following physical exercise, which may be relevant for metabolic health, although further studies are needed to determine the clinical implications of changes in essential metals such as manganese. While a 14-week combined exercise program has also reported reductions in toxic metals,^8^ the present data indicate a potentially greater reduction, possibly due to the use of high-intensity training. However, due to the lack of a control group and the small sample size, these findings should be interpreted with caution. Future randomized and controlled studies with larger populations are needed to confirm and expand on these results.

## Ethical approval

The study was approved by the Research Ethics Committee of the School of Physical Education and Sport at the University of São Paulo, with registration number CAAE 37,573,114.6.0000.5659. All participants signed the informed consent form to participate in the research.

## Data availability

Data will be made available upon request to the corresponding author.

## Authors’ contributions

GSR and NYN, Designed research; GSR, NYN, JALR, LMD, JB, IHYN, Conducted research, provided essential reagents; ACSS, FBJ, CBN, LMW, JB, CRBJ, Analyzed data; and GSR, NYN, JALR, LMD, IHYN wrote the paper GSR, NYN, JALR, JB, LMD, IHYN, ACSS, FBJ, CBN, LMW, CRBJ had primary responsibility for final content. All authors read and approved the final manuscript.

## Funding

This work was supported in part by a grant from Fundação de Amparo à Pesquisa do Estado de São Paulo (FAPESP) (23/08249-0).

## Conflicts of interest

The authors declare no conflicts of interest.
